# Tear levels of neuropeptides increase after specific allergen challenge in allergic conjunctivitis

**Published:** 2011-01-07

**Authors:** Marta Sacchetti, Alessandra Micera, Alessandro Lambiase, Stefania Speranza, Flavio Mantelli, Girolamo Petrachi, Sergio Bonini, Stefano Bonini

**Affiliations:** 1 Department of Ophthalmology, University of Rome Campus Bio-Medico, Rome, Italy; 2 IRCCS GB Bietti Eye Foundation of Rome, Rome, Italy; 3 Arma dei Carabinieri Headquarter of Rome, Rome, Italy; 4 Department of Medicine, Second University of Naples, Naples, Italy

## Abstract

**Purpose:**

Growing evidence is showing a role of neurogenic inflammation in allergic reactions, with sensory and autonomic nerve fibers releasing neuromediators, which may actively participate in the allergic inflammatory cascade. Although the cornea is the most densely innervated tissue of the human body, little is known on the role of neuromediators at the ocular surface. In this study, we aimed at evaluating the role of substance P (SP), calcitonine gene related peptide (CGRP), neuropeptide Y (NPY) and vasoactive intestinal peptide (VIP) in allergic reactions of the ocular surface.

**Methods:**

Fifteen patients with allergic conjunctivitis (6 female, 9 male, mean age 30±8 years) in non-active phase, and 10 age-matched healthy subjects were included in this study. The conjunctival provocation test (CPT) with allergen was performed in all allergic patients and in 5 healthy subjects. Tear samples were collected and the tear content of VIP, NPY, CGRP, and SP was measured by ELISA at baseline and after CPT. The Mann–Whitney U-test and Wilcoxon test were used to compare neuromediator tear levels.

**Results:**

No significant differences in neuropeptide tear levels were observed between healthy and allergic patients in non-active phase. CPT induced conjunctival hyperemia and itching in all allergic patients, while no reaction was observed in the control eyes and in healthy subjects. In allergic patients SP, CGRP, and VIP, but not NPY, were significantly higher after CPT as compared to baseline (SP: 3.9±1.3 ng/ml versus 5.8±1.1 ng/ml, p=0.011; CGRP: 5.5±2.3 ng/ml versus 7.3±2.7 ng/ml; p=0.002; VIP: 4±0.9 ng/ml versus 5.1±1.5 ng/ml, p=0.007). No significant changes were observed in the control eyes of allergic patients challenged with diluent and in healthy subjects after allergen provocation.

**Conclusions:**

Locally-released neuromediators may participate in modulating the allergic response of the ocular surface.

## Introduction

Growing evidence is showing that allergic reactions are influenced by the peripheral nervous system through the release of neuromediators including substance P (SP), neuropeptide Y (NPY), vasoactive intestinal peptide (VIP), and calcitonine gene-related peptide (CGRP) [1-5]. It has been widely demonstrated that SP, NPY, and CGRP are released from nerve endings at inflammatory sites and, more recently, a role of VIP at the sites of neurogenic inflammation has also been demonstrated in both animal models and humans [6-9]. In the airways, this neurogenic inflammation results in the release of neuropeptides by nerve endings, triggering the allergic reaction with bronchoconstriction, mucus secretion and mucosal hyperemia [10-12]. Specifically, it has been described that SP is released during allergen challenge in patients with allergic asthma and rhinitis, stimulating mucous secretion and histamine release by mast cells, enhancing migration and cytotoxic activity of eosinophils, and stimulating T-cell proliferation [13-17]. In line with this evidence, CGRP and VIP have been shown to induce vasodilatation and mucus secretion, to increase vascular permeability, and to stimulate leukocyte extravasation [18,19]. Moreover, VIP and NPY are known to play an immunomodulatory role by inducing T helper (Th2)- and inhibiting Th1-reactions [20,21].

While specific roles of different neuropeptides have been identified in allergic airway diseases, fewer data are currently available for ocular surface diseases. The ocular surface is a complex morphofunctional unit constituted by cornea, conjunctiva, lacrimal glands, and tear film [22]. During allergic inflammation, all these structures are activated and cross-talk through the release of cytokines and inflammatory mediators. A role of neuropeptides in this cross-talk has been recently suggested [1,4].

In line with this hypothesis, an increase of SP tear and plasma levels and an altered VIP and SP expression in the conjunctiva have been described in patients with vernal keratoconjunctivitis (VKC), a severe ocular allergic disease mainly affecting young boys [23-25]. Increased levels of SP have also been detected in tears of patients with seasonal allergic conjunctivitis, suggesting that SP may contribute to the pathogenesis and severity of disease [23]. Nevertheless, to date little is known on the role of neuropeptides in the ocular allergic reaction.

In this study we aimed at evaluating tear levels of SP, CGRP, NPY, and VIP in healthy and allergic subjects at baseline and after conjunctival allergen provocation test (CPT) [26].

## Methods

Fifteen asymptomatic patients previously diagnosed with allergic conjunctivitis (6 females, 9 males, mean age 30±8 years) and 10 healthy subjects (3 females, 7 males, mean age 30±15 years) were included in the study. The study was performed following the tenants of the Declaration of Helsinky. Informed consent was signed by all participants and the study was approved by the internal review board. Demographic and clinical characteristics of the patients in this study are summarized in Table 1.

**Table 1:** Demographic and clinical characteristics of patients with allergic conjunctivitis included in the study.

**Patients (n)**	**15**
**Sex (n)**	
Male	9
Female	6
**Age (years)**	
Range	20–51
Mean±SD	30±8
**Atopic associated diseases (n)**	
Rhinitis	9
Asthma	2
Dermatitis	0
**Skin PRICK test (n)**	
*Graminacee*	11
*Parietaria*	8
*Dermatophagoides*	4
**Conjunctival provocation test (n)**	
*Graminacee*	7
*Parietaria*	5
*Dermatophagoides*	3

Diagnosis of allergic conjunctivitis was based on clinical history, clinical examination, skin prick test and a positive conjunctival reaction following specific allergen challenge. Demographic, ophthalmological, and allergic history, as well as data on concomitant systemic and ocular medications used by the patients were collected at the inclusion visit. No signs and/or symptoms of allergic conjunctivitis were present at the time of inclusion in the study. Contact lens wearers and/or patients taking any topic and/or systemic anti-allergic drugs or steroids were excluded.

Skin prick test was performed in all allergic patients and healthy subjects with the same allergens used for CPT (*Dermatophagoides spp, Graminacee*, and *Parietaria*-Alk Abellò, Milan, Italy) to confirm the presence/absence of an allergic reaction. The allergen triggering the greatest allergic response in the skinprick test was chosen for the following CPT. CPT was performed according to a standardized procedure (Alk-Abellò, Milan, Italy). Briefly, increasing concentrations (0.001, 0.01, 0.1, 1.0, and 10.0 BU/ml) of antigen (*Dermatophagoides spp, Graminacee*, or *Parietaria)* were instilled into the conjunctival sac of the right eye every 15 min. Diluent alone was instilled in the left eye, used as control. Redness, itching and tearing were scored from 0 to 3 (0=absent, 1=mild, 2=moderate, 3=intense) and the sum was defined as the total CPT score (from 0 to 9). When a total CPT score ≥3 (with a score of conjunctival hyperemia of at least 2 and itching score of at least 1) was reached, allergen provocation was discontinued and the reaction was considered positive. The threshold dose was defined as the minimum allergen concentration required to obtain a positive reaction to CPT.

Five healthy patients were also challenged with *Dermatophagoides sp* (n=1) *Graminacee* (n=2), and *Parietaria* (n=2) and used as controls for non specific reactions caused by the allergen extract.

Tear samples were collected in allergic patients 1 h before CPT and 10 min following a positive CPT response, and in healthy subjects after the highest provocation dose [27]. All samples were collected in both eyes without previous topical anesthesia, as previously described. Briefly, dry Sharp-tip Microsponges (Alcon Lab. Inc., Fort Worth, TX) were inserted at the same time in the inferior conjunctival fornix, removed after 30 s and then immediately immersed in a 1.5 ml vial containing 50 μl of tissue protein extractor solution (TPER; Pierce, Rockford, IL) with cocktail protease inhibitors (Pierce). Microsponges were then centrifuged at 20,000× g relative centrifugal force for 3 min to recover tears [28]. The protein profile of tears collected was recorded according to the A280 program (NanoDrop Technology, Wilmington, DE) . Specific ELISA (Phoenix Pharmaceuticals Inc. Burlingame, CA; detection limit: 0.09 ng/ml NPY, 0.07 ng/ml SP, 0.28ng/ml CGRP 0.12 ng/ml VIP) were performed on extracted proteins to quantify the amount of neuropeptides, according to the manufacturer’s instructions.

The non-parametric Mann–Whitney U-test was used to compare tear levels of neuropeptides between allergic and healthy patients. Wilcoxon rank test was used to compare CPT scores and neuropeptide levels before and after CPT with allergen and with diluent. Neuropeptide levels at baseline and after challenge were also compared between the groups challenged with allergen and with diluent alone. (SPSS 15, Chicago, IL) Data are presented as mean±standard deviation (SD) from mean and a p value <0.05 was considered statistically significant.

## Results

**Figure 1:**
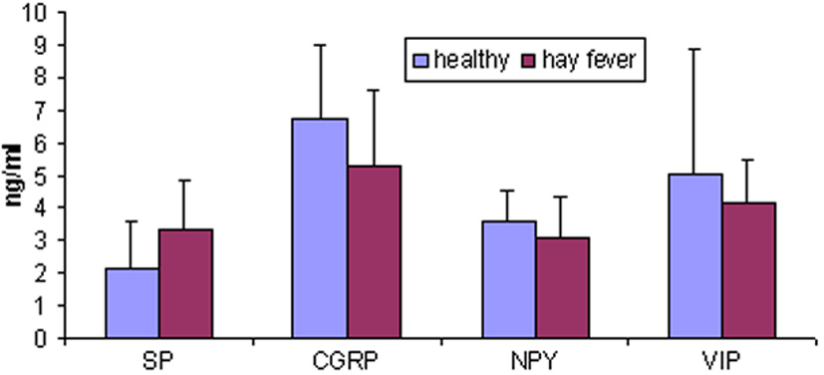
SP, CGRP, NPY, and VIP are present in tear fluid of healthy subjects and their values do not differ from those of allergic patients in non-active phase.

All neuropeptides were detected in tears of healthy subjects: SP=2.1±1.4 ng/ml; CGRP=6.7±2.3 ng/ml; NPY=3.5±1 ng/ml; VIP=5±3.8 ng/ml. No significant differences in neuropeptide tear levels were observed between healthy and allergic patients at baseline: SP=3.4±1.5 ng/ml; CGRP=5.3±2.3 ng/ml; NPY=3.1±1.3 ng/ml; VIP=4.1±1.4 ng/ml (Figure 1).

All allergic patients (100%) showed positive conjunctival reaction to CPT at a mean threshold dose of 1.7±3.1 BU/ml, evidenced by a significant increase of total CPT score (0 versus 3.9±1; p=0.001), itching (0 versus 1.5±0.6; p=0.026), and hyperemia (mean: 0 versus 2.2±0.5; p=0.023). No significant changes in tearing were observed (mean: 0 versus 0.2±0.4; p=NSS). No conjunctival reaction was observed in the control eyes challenged with vehicle (diluent) alone and in all the healthy subjects challenged with allergen.

**Figure 2:**
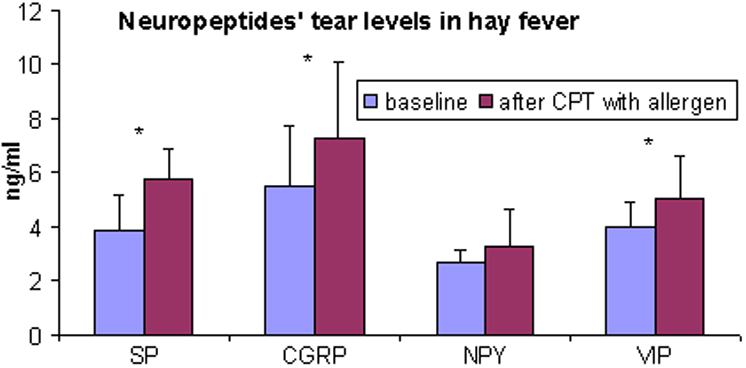
In allergic patients SP, CGRP, and VIP but not NPY tear levels significantly increase after a positive CPT.

Mean tear levels of SP, CGRP, and VIP were significantly higher after CPT (SP=3.9±1.3 ng/ml versus 5.8±1.1 ng/ml, respectively, p=0.011; CGRP=5.5±2.3 ng/ml versus 7.3±2.7 ng/ml; p=0.002; VIP=4±0.9 ng/ml versus 5.1±1.5 ng/ml, p=0.007). NPY tear levels showed no significant changes before and after CPT as compared to baseline values (2.7±0.4 versus 3.3±1.3 ng/ml, respectively, p=NSS; Figure 2). As compared to baseline, substance P was increased in 100% of the tear samples taken after allergen challenge, CGRP in 80%, VIP in 86%, and NPY in 62%. No significant differences in SP, CGRP, NPY, and VIP tear levels were detected in the control eyes before and after challenge with diluent (SP=4±1.2 ng/ml versus 4.8±2.2 ng/ml, respectively, p=NSS; CGRP=5.9±2.8 ng/ml versus 6.6±2.7 ng/ml; p=NSS; NPY=3.3±1.8 ng/ml versus 3.4±2.3 ng/ml, p=NSS; VIP=4.6±1.8 ng/ml versus 4.8±2.6 ng/ml, p=NSS). No significant changes were observed at baseline and after CPT in SP, CGRP, NPY, and VIP tear levels between eyes challenged with allergen and eyes challenged with diluent.

Substance P, CGRP, NPY, and VIP tear levels in healthy subjects showed no changes before and after CPT (SP=3.1±0.5 ng/ml versus 3.4±1.5 ng/ml, respectively, p=NSS; CGRP=6.8±3.7 ng/ml versus 7±4 ng/ml; p=NSS; NPY=3.6±1 ng/ml versus 3.6±0.8 ng/ml, p=NSS; VIP=5.4±4.1 ng/ml versus 5.4±2.9 ng/ml, p=NSS).

## Discussion

In this study we demonstrate that neuromediators are normally present in tears of healthy subjects, and that SP, CGRP, and VIP are increased after conjunctival allergen challenge in allergic patients.

The presence of neuromediators in normal conditions indicates that they may play a role in maintaining the ocular surface homeostasis. In line with this hypothesis, evidence from recent studies indicates that neuropeptides are involved in regulation of tear production from lacrimal grand and mucous secretion from conjunctival goblet cells [29]. Moreover, neuropeptides are also known to cooperate with innate immunity for the protection of ocular surface structures from pathogens and mechanical damage [22,30].

In this study we also show that SP, CGRP, and VIP are normally present in tears of allergic subjects and increase after conjunctival allergen challenge. It is unclear whether these changes represent a pathogenic mechanism of the allergic reaction or a mere epiphenomenon of the disease. Previous data from other groups support the hypothesis that changes in neuropeptide levels are involved in the pathogenesis of the allergic reaction. Specifically, an increase of SP tear and plasma levels has been described in patients with VKC [24] and increased SP tear levels were also demonstrated in patients with seasonal allergic conjunctivitis [23]. Our results show a significant increase of SP, CGRP, and VIP tear levels after challenge with allergen –but not with diluent– and no significant changes at baseline and after challenge between CPT eyes and control eyes. The similarity of mean neuropeptide levels before and after challenge with allergen and with diluent, apparently in contrast with our results showing a significant increase in the CPT group only, may be explained by the high variability in tear neuropeptide levels observed among individuals. However, using a “paired Wilcoxon Rank test” for statistical analysis –which analyses the magnitude of the difference in paired samples– allowed us to convincingly demonstrate the increase of SP, CGRP and VIP tear levels after CPT only in eyes challenged with allergen.

In our study, no differences were found in SP, CGRP, NPY, and VIP tear levels of asymptomatic patients with allergic conjunctivitis and healthy non-allergic subjects. A possible explanation to this finding is that all allergic patients included in our study were selected for being in a non-active phase –out of allergy season–, with no active signs and symptoms of allergic conjunctivitis at the time of inclusion. However, when these allergic patients were challenged with allergen by CPT, they showed a conjunctival allergic reaction characterized by itching and redness, paralleled by an increase of neuropeptide tear levels. Based on these results, we hypothesize conjunctival itching, hyperemia and chemosis during the allergic reaction could be influenced by locally released neuromediators. In line with this hypothesis, SP is believed to induce histamine release and mucus secretion, and to increase vascular permeability, while CGRP and VIP may exert blood vessel vasodilatation, leukocyte extravasation and cytokine secretion. Our findings are in line with evidence demonstrating an increase of SP, CGRP, and VIP after nasal allergen challenge and in airway hyperresponsiveness [16,31,32]. In our study, NPY was not significantly increased after conjunctival allergen challenge, suggesting a different role of this neuromediator in the ocular surface immune reaction. The role of NPY in allergic reaction is still largely debated. Previous studies showed that NPY induces vasoconstriction and that local pre-treatment with exogenous NPY in a nasal allergen challenge leads to a reduction of both nasal obstruction and mucus secretion, suggesting a protective effect of NPY on both vasodilation and secretory responses.

In conclusion, our study shows that neuropeptides are present in tears and may play an active role in both healthy and allergic conjunctiva. Locally released neuropeptides act directly on the epithelia, blood vessels and immune cells, potentially modulating protective and inflammatory responses. Our preliminary data showing an imbalance of tear neuropeptides during allergic reactions of the ocular surface requires further studies to clarify the specific role of individual neuropeptides in healthy conditions and in the mucosal response at different organ sites.
